# A Cross-Sectional Study of Factors Associated With Hospital Meal Choice in Medical Facilities in Hanoi, Vietnam

**DOI:** 10.7759/cureus.84462

**Published:** 2025-05-20

**Authors:** Reimi Shono, Shinji Nakahara, Kenji Toyama, Ikuko Gomi, Linh T Nguyen, Huong Lan T Nguyen, Fumiya Miyoshi, Aya Kuchiba, Haruhiko Inada, Huong T Le, Teiji Nakamura

**Affiliations:** 1 Nutrition, Graduate School of Health Innovation, Kanagawa University of Human Services, Kawasaki, JPN; 2 Emergency Medicine, Graduate School of Health Innovation, Kanagawa University of Human Services, Kawasaki, JPN; 3 Nutrition, Nara Women’s University, Nara, JPN; 4 Nutrition, Faculty of Health and Social Services, Kanagawa University of Human Services, Yokosuka, JPN; 5 Nutrition, School of Preventive Medicine and Public Health, Hanoi Medical University, Hanoi, VNM; 6 Nutrition and Dietetics, Hanoi Medical University Hospital, Hanoi, VNM; 7 Nutrition, Institute for Preventive Medicine and Public Health (IPMPH) Hanoi Medical University, Hanoi, VNM; 8 Nutrition, Saint Paul Hospital, Hanoi, VNM; 9 Nutrition, Nippon Telegraph and Telephone Corp. (NTT) Medical Center Tokyo, Tokyo, JPN; 10 Biostatistics, Graduate School of Public Health, Teikyo University, Tokyo, JPN; 11 Epidemiology/Public Health, Graduate School of Medicine, The University of Tokyo, Tokyo, JPN; 12 Nutrition, Kanagawa University of Human Services, Yokosuka, JPN

**Keywords:** hospital food service, hospitalized patients, inter-hospital differences, low- and middle-income countries (lmics), malnutrition

## Abstract

Objective: Although hospital food services are an important component of nutrition management for hospitalized patients, most hospitalized patients in low- and middle-income countries (LMICs) do not take hospital meals. This exploratory analysis aimed to determine the factors associated with the selective intake of hospital meals by hospitalized patients in Hanoi, Vietnam.

​Methods: This cross-sectional study collected personal attribute data from a survey of inpatient nutritional status at six hospitals in Hanoi conducted between 2018 and 2019, and hospital attribute data collected in 2022. Logistic regression models were used to analyze the association between individual variables and hospital meal intake; the hospital ID dummy variable was included in the model to obtain a coefficient adjusted for individual-level variables for each hospital. The associations between the coefficients and hospital-level characteristics were determined graphically because the limited number of hospitals did not allow us to statistically analyze them.

​Results: Of the 748 participants analyzed, 194 (25.9%) consumed hospital meals. A multivariable logistic regression analysis with hospital meal intake as the dependent variable and individual-level variables as the independent variables revealed that dietary changes before admission and disease type were associated with hospital meal intake. However, when introducing the hospital ID dummies, only the hospital dummy showed a significant association; individual-level factors did not show associations. The associations between hospital dummy coefficients and hospital characteristics were unclear.

​Conclusions: There were significant inter-hospital differences in the intake of hospital meals. Further research is needed on the factors influencing hospital meal intake.

## Introduction

Malnutrition in hospitalized patients is a global challenge regardless of the country’s economic situation, reportedly occurring in 20% to 50% of hospitalized patients [1,2]. Malnutrition and inadequate dietary intake during hospitalization are independently associated with poor prognoses and in-hospital mortality [2-4]. Therefore, properly managed hospital meals have been positioned as part of nutritional management for hospitalized patients [5].

In low- and middle-income countries (LMICs) with subpar nutrition management and hospital food service systems and high malnutrition prevalence, malnutrition in hospitalized patients is a greater challenge than in high-income countries (HICs). A systematic review of the nutritional status of hospitalized patients in Northeast and Southeast Asia showed that the prevalence of malnutrition exceeds 40% [6]. A study in Vietnam showed 34.1% malnutrition among adult hospitalized patients [7].

Despite the need for nutritional management, most hospitalized patients in LMICs eat meals brought from outside the hospital as hospital meals are often not an option [7]. Many hospitals do not provide meals, and even when they do, they do not have enough capabilities to serve all the patients. In Vietnam, 60% of patients consume meals prepared by family or delivered meals, with only those with special nutritional needs, such as postoperative gastroenterology patients, receiving hospital meals [8]. In contrast, in HICs like Japan, patients are required to eat hospital meals as part of treatment. In both LMICs and HICs, there has been little or no choice, and research mainly focuses on the amount of meal intake [9,10]. The only study on hospital meal choices in developing countries looked at adult patients’ dietary patterns but did not explore the reasons for their choice of hospital meals [11].

The recent establishment of hospital nutrition departments in some LMICs has introduced the option of eating hospital meals. In 2013, Hanoi Medical University launched a clinical dietitian training program, placing trained dietitians in hospitals across Vietnam as part of government policy to improve nutrition management for hospitalized patients [11]. However, in countries like Vietnam, patients are not required to eat hospital-provided meals. These recent changes have highlighted the need for research on factors influencing inpatients’ choice of hospital meals, a topic previously unexplored when no choice existed.

Therefore, this study aims to determine the selective intake of hospital meals by hospitalized patients in Hanoi, Vietnam, and to conduct an exploratory analysis to determine factors associated with the selection of hospital meals. Understanding the current dietary intake situation of hospitalized patients and factors associated with choosing hospital meals would be useful in improving nutrition management of hospitals in LMICs. 

## Materials and methods

Study design

This study is a secondary analysis of data obtained from a prospective observational cohort study conducted in Hanoi, Vietnam. The original study aimed to investigate the associations between nutritional status at hospital admission and outcomes (length of hospital stay and disease status at discharge) among inpatients aged 18 to 60 in eight public hospitals [12]. In the original study [12], data collection was conducted at admission and discharge. The admission data included patient characteristics and meal choices. The present study conducted cross-sectional analyses using only the admission data. The present study was approved by the Research Ethics Review Committee of Kanagawa University of Human Services (Decision Notice No. 71-7) and the Research Ethics Committee of Hanoi Medical University Hospital (Decision No. 35). Written informed consent was obtained from each patient. 

Study settings

In Vietnam, public hospitals serve most of the population and are categorized into central, provincial, and district levels [13]. Central and provincial hospitals provide specialized tertiary care to patients referred from district hospitals. District hospitals provide secondary-level care to patients referred from primary-care-level facilities [14, 15]. Public health insurance coverage is high in Vietnam. However, the insurance does not cover the hospital meal cost [11]. Since 2012, the Ministry of Health in Vietnam has been promoting the establishment of nutrition departments within hospitals to oversee meal services and nutritional management for patients, as well as the deployment of registered dietitians in each hospital [8]. Usually, attending physicians decide the type of therapeutic meal according to the patient’s disease conditions. However, patients are currently allowed to decide whether to take hospital meals; therefore, many bring food from outside. There is a social norm that family members should prepare meals for hospitalized patients.

Data collection in the original study

**Figure 1:**
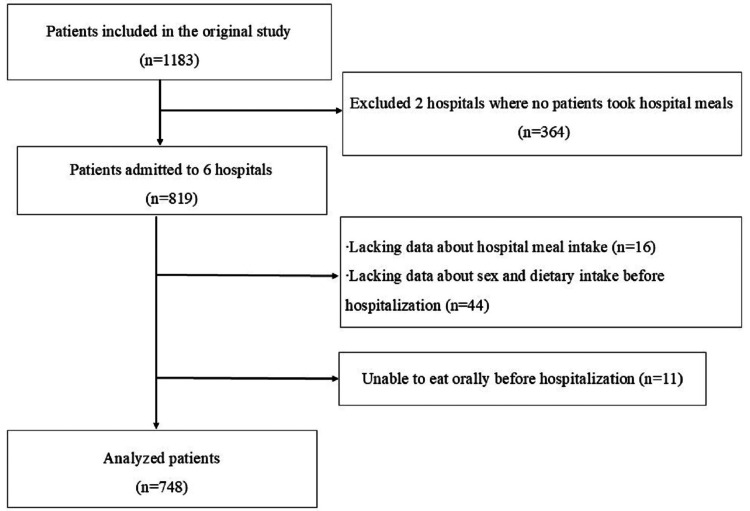
Flowchart on participant selection

The original study [12], the data source of the present study, was conducted in eight public hospitals in Hanoi, Vietnam: three central-level, one provincial-level, and four district-level hospitals. These facilities were chosen because of their wide variety of patients and easy accessibility for the researchers who conducted this study. The study was conducted from September 1, 2018, to March 31, 2019, in the central and provincial-level hospitals and from July to November 2019 in the four district hospitals (Figure 1). The study included all the participants who met the inclusion criteria (n = 1,183) of hospitalization between three days and four weeks, aged 18 to 60 years, dietary intake within 48 hours of admission, and the ability to stand and speak. The exclusion criteria were those who did not consent to the study, those not allowed to participate in the study by the attending physician, those who could not speak Vietnamese, those who had infectious diseases, those admitted to the intensive care unit or who were bedridden, and those not yet assigned a hospital bed (bed allocation was defined as “hospitalized”).

Data collection in the original study was conducted prospectively via a survey (see Appendix A) [12]. Research assistants transcribed the date of admission, sex, age, primary diagnosis, and laboratory data from the medical record within two days of admission. The assistants interviewed the patients regarding weight change before admission in the past six months and in the past two weeks, or loose clothing worn over the past three months if they had not been weighed; dietary intake status before admission (no change/increase, suboptimal solid diet, liquid diet, and could not orally eat); gastrointestinal symptoms (nausea, vomiting, diarrhea, and anorexia in the two weeks preceding admission); and current dietary status (taking hospital meals or not). They performed a physical examination to assess subcutaneous fat, muscle mass, and edema. They also measured weight and height using a portable height scale (Seca 213; Seca GmBH & Co., Hamburg, GER) and a weight scale (BC-758; Tanita Corporation, Tokyo, JPN); two measurements were averaged, respectively. After discharge, the assistants collected information on prognostic status from the post-discharge medical records. The research assistants were employees of the nutrition department of the respective hospitals. Skilled Vietnamese dieticians trained the assistants in data collection and physical measurements before the study. 

Participants 

In this secondary analysis, data from six of the eight hospitals surveyed in the original study [12] were used; two hospitals were excluded because they had no patients consuming hospital meals. Such hospitals did not provide hospital meals and were unsuitable for analyzing factors associated with patients' choices of hospital meals. Of the six hospitals, three, one, and two were central, provincial, and district hospitals, respectively. Of the 1183 patients included in the original study, 819 were registered from the six hospitals. Patients with missing data on the selection of hospital meals (n=16) and those with missing data on sex and dietary intake status before admission (n=44) were excluded. Those who could not eat orally before admission were also excluded because they had no choice of eating hospital meals (n=11). The final sample for the analysis comprised 748 patients (Figure 1). 

Analyzed data and the primary endpoint

The present study features the following data extracted from the original study [12]: sex, age, BMI, primary diagnosis, current diet, weight change, dietary intake before hospitalization, and digestive symptoms. The hospital attributes as of 2019 were inquired from the nutrition department of respective hospitals or collected from official information published by each hospital by a Vietnamese co-researcher in June 2022. This included the number of beds, cost of standard meals, years since the nutrition department was established, number of staff in the nutrition department, and the introduction of charity meals (private organizations or companies often provide charity meals in the form of boxed lunches that are prepared and served in the cafeteria). To analyze each facility’s geographic location, we identified their addresses from the information on their official websites. The primary endpoint was whether hospitalized patients consumed hospital meals or meals from outside at the time of the interview.

Individual-level explanatory variables

Patient age was classified into 18-24, 25-34, 35-44, 45-54, and ≥ 55 years. The BMI at admission was categorized into <18.5 kg/m², 18.5-24.9 kg/m², and ≥25 kg/m² according to the Japan Society for the Study of Obesity categorization [16]. Regarding weight change, those who reported “weight loss” in the past six months or the past two weeks or whose clothes became “a little” or “very” loose in the past three months were defined as having “weight change.” Those who reported none of the above were defined as having “no weight change.

Regarding the diet change before hospitalization, those who responded “suboptimal solid diet or liquid diet” were defined as “changed.” Those who responded “no change/increase” were defined as “no change.” For gastrointestinal symptoms, “symptomatic” was defined as the presence of any of the symptoms indicated in the interview. Otherwise, they had “no symptoms.” The primary diagnosis was coded using the International Statistical Classification of Diseases and Related Health Problems (ICD-10) [17] and categorized at the ICD chapter level. The categories with small numbers were combined as “other diseases.”

Hospital-level explanatory variables

The hospital type was categorized into core hospitals (central and provincial-level hospitals) and district hospitals. The following variables were dichotomized at the median: the number of beds, the cost of standard meals as of 2019, the number of staff in the nutrition department per 100 beds, the years since the establishment of the nutrition department to 2019, the distance from each hospital to Hanoi Station using Google Maps, and the median BMI for each hospital (calculated as an indicator of the nutritional status in the hospital as a whole). Charity meals provided by charitable organizations in any form were classified as either yes or no. 

Statistics analysis

Patients with missing data were excluded from the analysis because of their small number. Descriptive statistics on the participants’ and hospital characteristics were presented using proportions and median (interquartile range). These variables were those we assumed to be related to the selection of hospital meals. The strength of the association between the characteristics and the selection of hospital meals was confirmed using Cramer’s V, which indicates the strength of associations between categorical variables as a coefficient between 0 and 1.

Then, logistic regression analysis was performed with the dichotomous dependent variable of hospital meal selection (selected hospital meals or not) to calculate odds ratios (ORs) with a 95% confidence interval (CI). Logistic regression models were used because the outcome variable was binary. Additionally, while we acknowledge that using multilevel modeling is preferable because it enables us to simultaneously adjust for group- and individual-level variables and accurately estimate their regression coefficients, we were unable to do so in this study. This is because, in multi-level analyses, a small number of groups would result in unstable or biased estimations, and at least 20-30 groups are required for stable estimations [18]. Therefore, the following analysis procedures were used.

First, univariate analyses were performed to estimate crude ORs for the individual-level explanatory variables and the hospital ID dummy variable. Second, after adjusting for the other variables, a multivariable analysis including all individual-level explanatory variables was performed to determine their independent associations with the selection of hospital meals (model 1). Third, a multivariable analysis (model 2) was performed with hospital ID dummies alongside the individual-level variables without a constant term to obtain an estimate of the fixed effects of each hospital rather than random effects, which are usually estimated in multi-level models [19]. It was the hospital-level indicator of the selection of hospital meals after adjusting for the individual-level variables. The logistic regression models were assessed using the deviance (-2 log-likelihood), Hosmer-Lemeshow test, and areas under the receiver operating characteristic curves, which evaluate overall model fit, local model fit, and discriminative ability, respectively.

Then, scatter plots of the hospital-level coefficient estimates and the hospital-level variables were created to assess their associations visually. Preferably, the hospital-level coefficients would have been used to perform multiple linear regression analysis with the hospital-level explanatory variables, an introductory approach to multilevel analysis. However, it was impossible due to the small number of hospitals.

Because the sample size was predetermined in this study, which used existing data, the minimum detectable effect size was calculated based on the given individual-level sample size using G*Power (Ver. 3.1.9.7) [20]. The minimum detectable OR in a logistic regression analysis with individual-level variables was 1.56. This was based on the given sample size (n=748), a binary explanatory variable having the same probability of taking either value, power of 80%, a two-sided 5% significance level, and a null hypothesis that 26% of the patients take hospital meals in both groups defined by the binary explanatory variable. An odds ratio of 1.5 means that the odds of choosing hospital meals are 1.5 times greater when a certain factor is present than when it is absent, which is a clinically significant effect size. The EZR version 1.55 (Jichi Medical University Saitama Medical Center, Saitama, JPN) was used as the statistical analysis software [21]. This software is a modified version of R Commander, designed to run R and be used in Japanese.

## Results

Most participating hospitals were core hospitals and had charity meals (Table 1). Their number of beds varied considerably (116-3600, median 790). The hospital meal prices did not differ considerably (median 26,500 Vietnamese dong (VND); about US$ 1.1). The age of the nutrition department ranged from two to 18 years (median 7). The number of staff per 100 beds ranged from 0.6 to 3.4 (median 0.9). The distance from the hospital to the Hanoi train station ranged from 1.2 to 9.1 km. The median BMI of patients at each hospital ranged from 21.0 to 22.8 kg/m2 (median 21.9 kg/m2).

**Table 1:** Hospital characteristics (n=6) VND: Vietnamese dong

Characteristics	N (%)/Median (minimum-maximum)
Hospital type	
Core	4 (66.7)
District	2 (33.3)
Charity meal	
Yes	4 (66.7)
No	2 (33.3)
Meal cost (VND)	26,500 (24,000−35,000)
Number of beds	790 (116−3600)
Number of staff per 100 beds	0.9 (0.6−3.4)
Time since establishment of nutrition department (years)	7 (2–18)
Distance from Hanoi Station (km)	3 (1.2−9.1)
Median BMI (kg/m^2^)	21.9 (21.0−22.8)

Also, 194/748 (25.9%) participants consumed hospital meals (Table 2). About half were female. Most were aged ≥45 years, had normal nutritional status, had weight loss before admission, had no digestive symptoms, and had no dietary intake change. The most common primary diagnosis for hospitalization was digestive system diseases (22.6%), followed by neoplasms (22.5%). These characteristics were not associated with the selection of hospital meals (Cramer’s V <0.1).

**Table 2:** Patient characteristics by meal choice (n=748) ^a^ Symptoms, signs, and abnormal clinical and laboratory findings, not elsewhere classified. The strength of the association between the characteristics and the selection of hospital meals was confirmed using Cramer’s V, which indicates the strength of associations between categorical variables as a coefficient between 0 and 1.

Characteristics	Meal selection		Cramer’s V
	Hospital meals	Non-hospital meals	
	N (%)	N (%)	
Sex			0.08
Male	110 (29.5)	263 (70.5)	
Female	84 (22.4)	291 (77.6)	
Age Category			0.03
≤24	13 (23.2)	43 (76.8)	
25–34	30 (25.6)	87 (74.4)	
35–44	49 (27.7)	128 (72.3)	
45–54	52 (24.6)	159 (75.4)	
≥55	50 (26.7)	137 (73.3)	
Nutritional status (BMI kg/m^2^)			0.01
Normal weight (BMI 18.5–24.9)	144 (26.2)	406 (73.8)	
Underweight (BMI<18.5)	21 (25.6)	61 (74.4)	
Overweight (BMI≥25.0)	29 (25.0)	87 (75.0)	
Weight change			<0.01
Weight decrease or loose clothing	128 (25.7)	371 (74.3)	
No change/Increase/Unknown	66 (26.5)	183 (73.5)	
Digestive symptoms			0.03
Yes	32 (23.0)	107 (77.0)	
No	162 (26.6)	447 (73.4)	
Diet: Before hospitalization vs. Usual			0.08
No change or increased intake	147 (24.3)	458 (75.7)	
Suboptimal solid or liquid	47 (32.9)	96 (67.1)	
Primary diagnosis			0.09
Digestive system	51 (30.2)	118 (69.8)	
Neoplasms	38 (22.6)	130 (77.4)	
Genitourinary system	19 (25.7)	55 (74.3)	
Endocrine, nutritional, and metabolic	17 (29.8)	40 (70.2)	
Not elsewhere classified^ a^	7 (14.9)	40 (85.1)	
Other diseases	62 (26.6)	171 (73.4)	

Most patients were hospitalized in core level hospitals with charity meals (meal cost <26,500 VND), <790 beds, more nutrition staff (≥0.9 per 100 beds), and a nutrition department with longer history (≥7 years), located far from the city center, and with higher median BMI (≥21.9) (Table 3). The presence of charity meals, higher meal costs, and a longer history of the nutrition department were slightly associated with a lower proportion of the selection of hospital meals (V >0.1). The other characteristics were not associated with the selection of hospital meals.

**Table 3:** Hospital characteristics by meal choice (n=748) VND: Vietnamese dong The strength of the association between the characteristics and hospital food intake was confirmed using Cramer’s V, which indicates the strength of associations between categorical variables as a coefficient between 0 and 1.

Characteristics	Hospital meals	Non-hospital meals	Cramer’s V
	N (%)	N (%)	
Hospital type			0.07
Core	160 (27.5)	422 (72.5)	
District	34 (20.5)	132 (79.5)	
Charity meal			0.21
Yes	90 (18.9)	385 (81.1)	
No	104 (38.1)	169 (61.9)	
Meal cost (VND)			0.13
<26,500	175 (28.7)	434 (71.3)	
≥26,500	19 (13.7)	120 (86.3)	
Number of beds			0.03
<790	105 (27.2)	281(72.8)	
≥790	89 (24.6)	273 (75.4)	
Number of staff per 100 beds			0.03
<0.9	89 (24.6)	273 (75.4)	
≥0.9	105 (27.2)	281 (72.8)	
Time since establishment			0.12
<7 years	108 (31.6)	234 (68.4)	
≥7 years	86 (21.2)	320 (78.8)	
Location of the hospital from Hanoi Station			0.08
＜3 km	88 (30.1)	204 (69.9)	
≥3 km	106 (23.2)	350 (76.8)	
Median BMI (kg/m^2^)			0.08
<21.9	74 (22.1)	261 (77.9)	
≥21.9	120 (29.1)	293 (70.9)	

In the univariate logistic regression analysis (Table 4), females were less likely than males to opt for hospital meals (OR=0.69 (95% CI, 0.49-0.96)); those with deteriorated food intake before hospitalization were more likely to opt for hospital meals than those with regular intake (OR=1.53 (95% CI, 1.03-2.2)); and those with “symptoms, signs, and abnormal clinical and laboratory findings not otherwise classified” were less likely to take hospital meals than those with digestive disease (OR=0.41 (0.17-0.96)). Hospital differences were also significant.

**Table 4:** Association between the selection of hospital meals and individual-level and hospital-level factors among hospitalized patients (n=748) *Statistically significant (p < 0.05) ^b^ Adjusted odds ratios for individual-level variables only ^c^ Adjusted odds ratios for individual-level variables plus hospital dummy variables H-L: Hosmer–Lemeshow; AUC: Area under the receiver operating characteristic curve Logistic regression analysis was performed with the selection of hospital meals as the dependent variable to calculate ORs with 95% CI. Multilevel analysis was preferred because of the two-level data structure (patients were nested in the hospitals), which was impossible due to the small number of hospitals. Therefore, univariate analyses; a multivariable analysis including all individual-level explanatory variables to determine their independent associations with the selection of hospital meals, adjusting for the other variables (model 1); and a multivariable analysis with hospital ID dummies alongside the individual-level variables without a constant term to obtain an estimated regression coefficient for each hospital (model 2), were performed.

Variables	Crude odds ratio (95% Cl)	Model 1^b^ adjusted odds ratio (95% Cl)	Model 2^c^ adjusted odds ratio (95% Cl)
Sex			
Female/Male	0.69 (0.49– 0.96)	0.71 (0.50–1.00)	0.85 (0.58–1.25)
Age (years)			
<24	1	1	1
24–34	1.14 (0.54–2.41)	1.29 (0.60–2.78)	1.12 (0.49–2.56)
35–44	1.27 (0.63–2.56)	1.42 (0.69–2.93)	1.51 (0.69–3.28)
45–54	1.08 (0.54–2.17)	1.07 (0.53–2.19)	1.11 (0.52–2.38)
≥54	1.21 (0.60–2.43)	1.29 (0.63–2.67)	1.68 (0.77–3.68)
BMI (kg/m^2^)			
Normal weight (BMI 18.5–24.9)	1	1	1
Underweight (BMI <18.5)	0.97 (0.57–1.65)	0.96 (0.55–1.68)	0.86 (0.47–1.57)
Overweight (BMI ≥25.0)	0.94 (0.59–1.49)	0.92 (0.57–1.49)	0.87 (0.51–1.47)
Weight change			
Weight loss/No weight loss	0.96 (0.68–1.35)	0.87 (0.61–1.25)	0.95 (0.63–1.42)
Digestive symptoms			
Yes/No	0.83 (0.54–1.27)	0.71 (0.45–1.15)	0.95 (0.56–1.63)
Dietary intake before hospitalization vs. usual			
No change or increase	1	1	1
Suboptimal solid diet or liquid diet	1.53 (1.03–2.26)	1.82* (1.17–2.81)	1.13 (0.68–1.88)
Disease			
Digestive system	1	1	1
Neoplasms	0.68 (0.42–1.10)	0.70 (0.42–1.15)	0.94 (0.53–1.67)
Genitourinary system	0.80 (0.43–1.48)	0.85 (0.45–1.59)	1.25 (0.62–2.54)
Endocrine, nutritional, and metabolic diseases	0.98 (0.51–1.89)	1.08 (0.55–2.13)	1.64 (0.78–3.45)
Symptoms, signs, and abnormal clinical and laboratory findings, not elsewhere classified	0.41 (0.17–0.96)	0.40* (0.16–0.98)	0.65 (0.25–1.72)
Other diseases	0.84 (0.54–1.30)	0.94 (0.59–1.49)	1.30 (0.78–2.16)
Hospitals			
Hospital 1	1		-0.85* (-1.67–-0.03)
Hospital 2	1.54 (1.02–2.32)		-0.42 (-1.28–0.44)
Hospital 3	0.11 (0.04–0.33)		-2.89* (-4.26–-1.52)
Hospital 4	0.02 (<0.1–0.12)		-5.06* (-7.20–-2.92)
Hospital 5	0.24 (0.14–0.43)		-2.4* (-3.38–-1.42)
Hospital 6	4.21 (1.76–10.10)		0.47* (-0.75–1.69)
-2 log-likelihood	−	836.54	693.25
H-L test AUC		Chi-squared = 5.3, df = 8, p-value = 0.727 0.60 (0.56–0.65)	Chi-squared = 3.8, df = 8, p-value = 0.875 0.78 (0.74–0.81)

In model 1, i.e., the multivariable model with only the patient-level variable, females, those with deteriorated food intake, and those with “symptoms, signs, and abnormal clinical and laboratory findings not otherwise classified” showed significant associations with the selection of hospital meals. When the hospital ID dummy variable was added in model 2 without a constant term, only the hospital ID showed significant associations. None of the individual-level variables showed significant associations. In both models, Hosmer-Lemeshow statistics were not significant (predicted and observed values were not different), and AUCs were larger than 0.6. In model 2, the AUC was larger and the deviance was smaller than in model 1.

**Figure 2:**
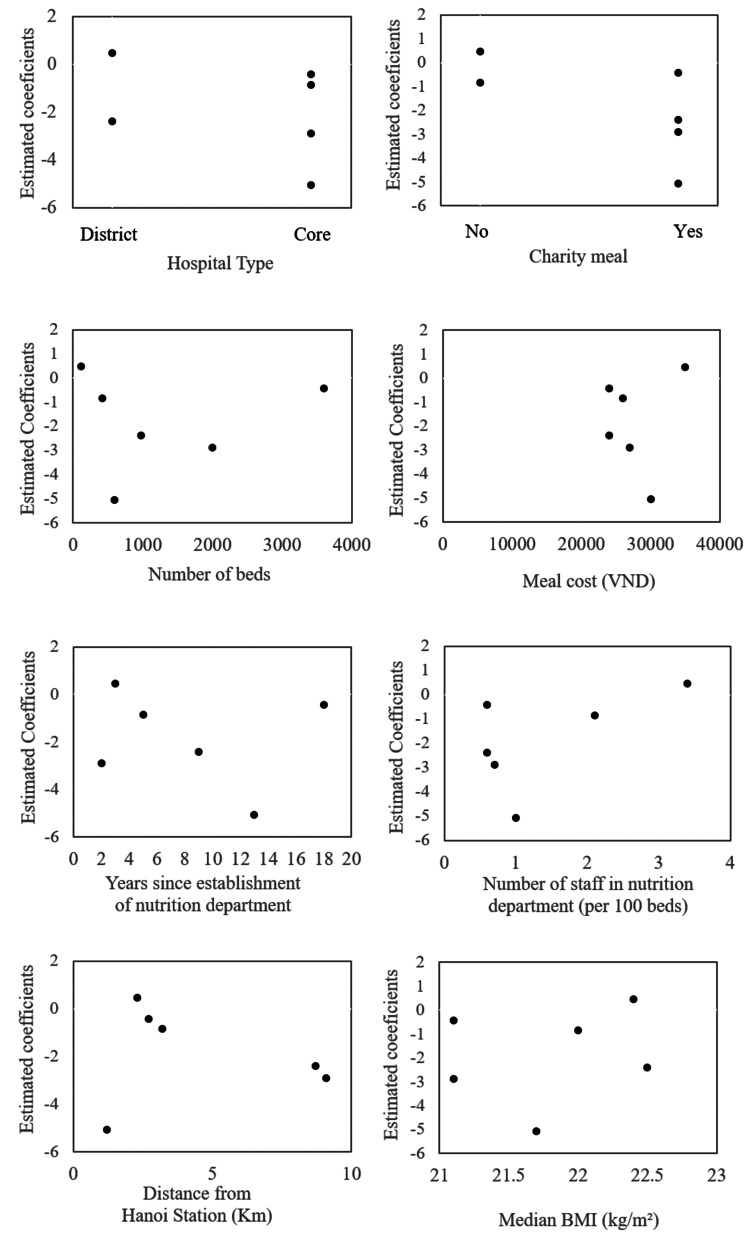
Scatterplots of estimated coefficients for hospitals and hospital type, charity meals, number of beds, meal cost, years since the establishment of the nutrition department, number of staff in the nutrition department, location, and median BMI The scatter plots of the hospital-level coefficient estimates and the hospital-level variables were created to assess their associations visually. VND: Vietnamese dong

Hospital ID coefficients in model 2 (indicating the selection of hospital meals after adjusting for individual-level variables) were plotted against hospital-level variables (Figure 2) to assess their associations visually. The selection of hospital meals was higher for district hospitals than for core hospitals and higher in the absence than in the presence of charity meals. However, the associations were unclear due to the small sample size. The other hospital-level characteristics did not show clear associations.

## Discussion

In this study, only a quarter of the hospitalized patients took hospital meals in facilities where food services are provided to inpatients. Additionally, in two of the eight hospitals in the original study, the data source of the present study, no patient took a hospital meal. Most ate food brought from outside the hospitals and were not supervised by nutrition experts concerning disease conditions. In the exploratory analysis to identify factors associated with the selection of hospital meals, a multivariable model with only individual-level factors showed that sex, dietary change before admission, and primary diagnosis were associated with the selection of hospital meals. However, when the hospital ID dummies were included in the model, they showed a significant association, and the significance of the individual-level factors disappeared. This result means that inter-hospital differences explain the patients’ decision to take hospital meals more strongly than individual-level factors. However, it was not possible to statistically identify specific hospital-level factors that explain the inter-hospital differences due to a lack of statistical power (the number of hospitals was insufficient).

To our knowledge, this is the first study to investigate factors associated with hospital meal intake. Numerous studies have investigated the nutritional status of hospitalized patients. Malnutrition has been reported to affect 20% to 50% of inpatients, regardless of a country’s economic status [1]. Additionally, a study conducted in Ho Chi Minh City, Vietnam, reported that 34.1% of participants were malnourished [11]. These findings are similar to our study, which reports a malnutrition prevalence of 26%, indicating that malnutrition among hospitalized patients is a global issue.

However, no prior studies investigating hospital meal choices exist. This is because eating hospital meals is the norm in HICs, and the importance of nutrition management through hospital meals is not well understood in LMICs. Therefore, previous studies in HICs focused on factors associated with the amount of food intake in the hospital, assuming that the patients eat hospital meals [2,9,10]. Studies in LMICs described nutrition status and dietary sources (bring-in or hospital meals) and the amount of food intake, but not the reasons for meal choices [11]. 

A prior study in Ho Chi Minh City reported that only 1.3% of hospitalized patients consumed hospital meals [11]. In contrast, the present study found the proportion to be 25.9%. In both studies, the proportion of patients consuming hospital meals fell short of 100%, while that of Hanoi is much higher than that of Ho Chi Minh. The differences between the studies could reflect the recent establishment of dietitian training courses and hospital nutrition departments in Vietnam. Investigating the causes of the differences could lead to effective intervention to increase the selection of hospital meals.

The results of this exploratory study may lead to future research to improve nutrient management in hospitalized patients in many LMICs where patients choose whether or not to eat hospital meals. However, such studies are not relevant in most HICs, where eating hospital meals is mandatory and bringing your own food is not allowed. Increasing the number of patients who eat hospital meals managed by a dietician would facilitate nutritional management during hospitalization. The hospital dieticians cannot monitor and adjust the calories and nutrients consumed when food is brought in. Such meals are not appropriate for patients. A Vietnamese study reported that the energy intake of hospitalized patients who ate bring-in meals was only 840 kcal/day [11].

Several limitations of this study should be noted. First, this study was a secondary data analysis, meaning the data were not collected to address its specific objectives. The original study, the data source of the present study, aimed to describe the nutritional status of hospitalized patients. Consequently, several potentially relevant variables that may influence hospital meal intake were not measured, i.e., the meal menu, sources of brought-in food, reasons for patients’ choice of “current diet,” and patients’ socioeconomic background. However, the unmeasured factors were unlikely to confound the findings because they are elements of hospital capacities or environments or unrelated to the hospital-level characteristics (e.g., socioeconomic background). 

Second, this study used cross-sectional data obtained at the time of patient admission; it was not possible to follow changes in dietary intake during hospitalization. Patients might have started consuming hospital meals during their hospitalization. However, this is quite unlikely, as most patients’ food choices depend on their preferences, cultural and social norms, and affordability, lacking appropriate nutrition management by dietitians in relation to disease conditions.

Third, the small sample size of hospitals prevented us from statistically examining the association between hospital-level factors and the selection of hospital meals using a multi-level analysis. Instead, we used a fixed effect model, which may overestimate the statistical significance. However, the ORs for hospital dummies were large enough that larger standard errors expected in a multilevel analysis would not change the results. Further studies are required to determine the hospital-level characteristics that are related to the choice of hospital meal intake.

Although this study could not examine specific factors at the hospital level, some possible explanations for the inter-hospital differences in hospital meal intake are discussed here to provide a basis for future research. First, factors that may reduce patients’ appetite may prompt them to choose food they are used to eating. The dining environment in the hospital, with various catheters, fluid bags, and equipment, may reduce patients’ appetite [22]. Food service quality also influences appetite, e.g., menu variety, appearance, taste, and meal temperature control using temperature-controlled wagons for transporting meals [23,24]. 

Second, the ability of the nutrition department to provide quality nutrition management may be relevant. Although some of the surveyed hospitals were comparable to hospitals in Japan regarding the number of staff per bed [25], the present study did not explore the staff qualifications and training contents. If the staff training is not appropriate, counting the number of staff is irrelevant. This study used the number of years since the establishment of the nutrition department as a proxy measure of the maturity of their nutrition management activities. However, the number of years does not determine the quality of care [26]. Conversely, a good relationship between patients and healthcare workers and good presentation of meals may improve patients’ appetite [22,27].

Third, economic factors may play important roles. The hospital meal cost, which is currently not covered by public health insurance in Vietnam, and charity meals may be associated with particularly low-income families. Charity meals, provided for free to support patients economically, are not prepared per patients’ clinical needs and have hygiene problems. If charity meals are associated with patients’ choice of not opting for hospital meals, this stance should be changed by providing financial support or health insurance to cover the cost of the hospital meal.

Fourth, the geographical situation may be associated with the selection of hospital meals. Patients have better access to restaurants and cafeterias near hospitals in the city center. However, the culture of bringing your own food may still be stronger in suburban areas. These factors were not appropriately reflected in the distance from the city center. Although a number of factors may play a role, cultural and social norms are likely the most influential. In Vietnam, it is considered the family's duty to care for the sick, including preparing meals. As a result, hospitalized patients are always accompanied by at least one family member, sometimes more, who provides meals for the patients. This practice is not unique to Vietnam but is also common in many other LMICs. The influence of cultural and social norms should be investigated by further research.

Fifth, the inter-hospital differences in hospital meal intake can be attributed to their large disparities in capacity to provide hospital meals. Some hospitals have large, well-equipped kitchens with sufficient staff, while others lack sufficient resources to provide food services. Notably, two hospitals without food services were excluded from the analysis. Despite the limitations and inability to identify specific factors related to the selection of hospital meals, inter-hospital disparities observed in the present study are likely a common challenge in LMICs with partially implemented hospital nutrition care systems.

Regarding the impact of the previously mentioned limitations on generalizability, the factors not measured in this study are either related to hospital capacity or environmental aspects or are independent of hospital-level characteristics, making it unlikely that they confounded the findings. Furthermore, while this study was unable to track changes in dietary intake during hospitalization, most patients consumed food brought from outside the hospital, suggesting that the likelihood of significant changes in dietary intake during hospitalization is low. Therefore, we consider that the findings of this study are not substantially restricted in terms of generalizability. Low- and middle-income countries require standardized nutrition management, including nutrition department competencies and kitchen capacity to serve all inpatients. Without a solid foundation for food services and nutrition management, patients’ choice of hospital meals would remain limited.

To address these disparities, legislation should be considered to standardize the hospital food service systems. Such legislation could require hospitals to set up complete food service systems and even mandate inpatients to consume hospital meals, acknowledging the challenge of changing behaviors based on cultural and social norms. The legislation could also require the health insurance to partially or fully cover the meal cost. 

In HICs, hospital meals are typically an integrated part of the hospital care system, often regulated by law. For example, in Japan, under the Medical Service Act enacted in 1948, complete hospital meal services and dietitians were legally positioned in hospitalized medical care. Before this period, patients brought their own food or prepared their meals in the hospital. Since 1950, hospitalized patients in Japan have been required to take hospital meals [28]. To alleviate the financial burden on patients, health insurance covers part of the meal cost.

Although the present study cannot draw definitive conclusions, the progress of nutrition management in HICs and the contrasting findings from Ho Chi Minh City highlight the need to integrate nutrition management into medical care. Policymakers and healthcare providers in Vietnam and other LMICs should address this need by institutionalizing and mandating the establishment of nutrition departments and ensuring the provision of affordable hospital meals. In Vietnam, however, dietitian training is still in its early stages, and many hospitals lack sufficient food supply capacity. Strengthening the dietitian training and certification system, along with increasing budgets for hospital food service facilities, is essential.

## Conclusions

Most hospitalized patients in the studied hospitals in Hanoi, Vietnam, did not consume hospital meals and ate food brought from outside, which was not supervised or adjusted by nutrition experts to meet disease conditions. The exploratory analysis could not identify specific individual-level or hospital-level factors associated with hospital meal selection but indicated a large inter-hospital variation. Although the findings cannot lead to decisive conclusions, possible factors that explain the variation include hospital food service capacity, interventions to address economic issues, and cultural norms surrounding family roles. Further research is needed on the factors that influence hospital meal selection; efforts should be made to standardize hospital nutrition management in LMICs. Policy measures such as mandating the provision of hospital meals, integrating dietitians into inpatient care, and including meal costs in health insurance coverage are critical to ensuring adequate nutritional support during hospitalization.

## Figures and Tables

**Figure 3:**
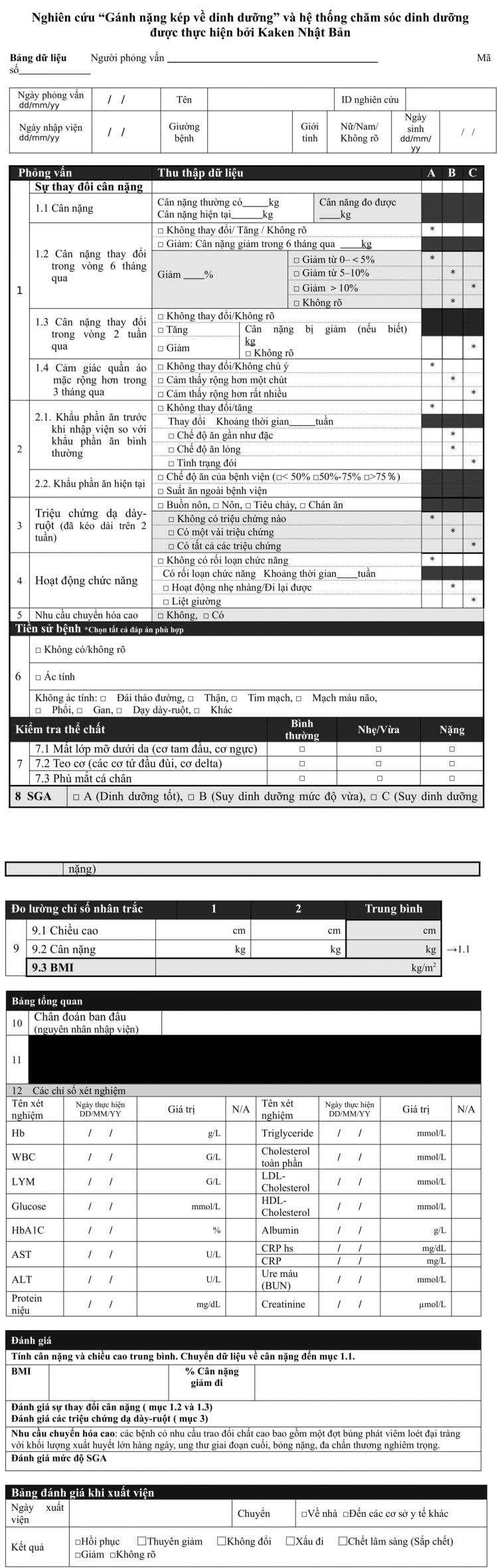
The original survey form in Vietnamese

**Figure 4:**
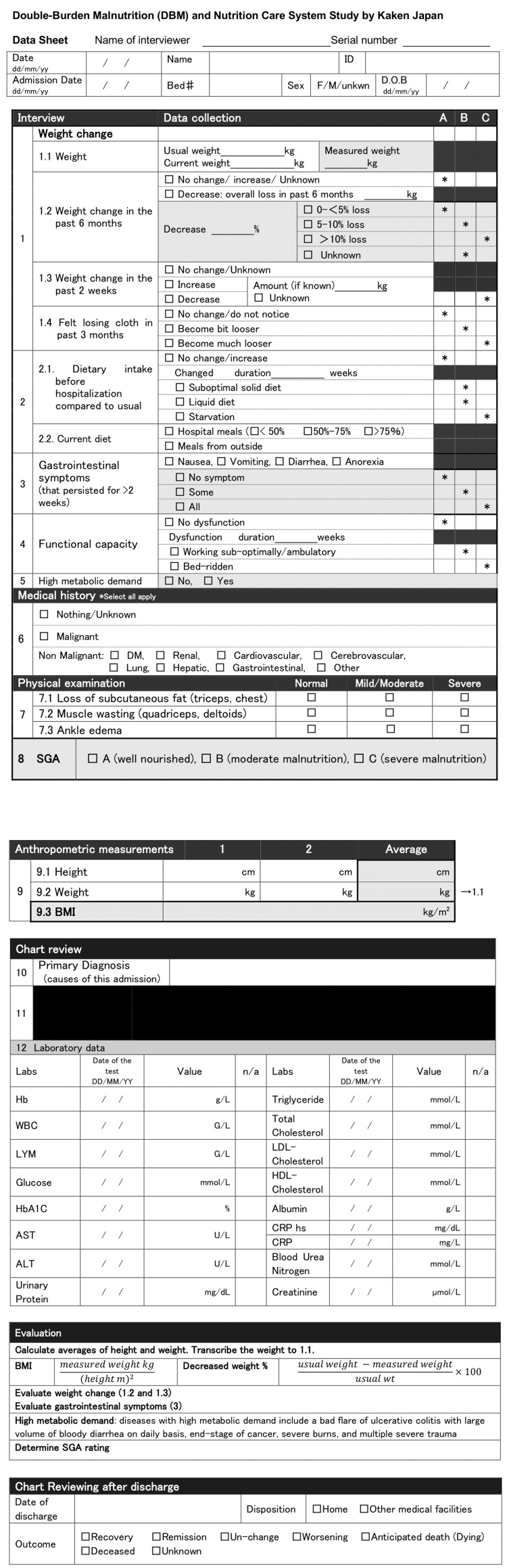
English translation of the survey form

## Appendices

Appendix A 

The survey forms that were used to collect patient information for the original study [12], on whose data the present study is based, are featured in Figures 3-4.
